# To Produce or to Survive: How Plastic Is Your Crop Stress Physiology?

**DOI:** 10.3389/fpls.2017.02067

**Published:** 2017-12-05

**Authors:** Ahan Dalal, Ziv Attia, Menachem Moshelion

**Affiliations:** Faculty of Agriculture, Food and Environment, The Robert H. Smith Institute of Plant Sciences and Genetics in Agriculture, The Hebrew University of Jerusalem, Rehovot, Israel

**Keywords:** canalization, coefficient of variation (CV), G × E interaction, phenotypic plasticity, QPT hierarchy, water relations

## Abstract

Abiotic stress causes major crop losses and is considered a greater challenge than biotic stress. Comparisons of the number of published articles and patents regarding these different types of stresses, and the number of commercially released crops designed to tolerate different types of stresses, revealed a huge gap in the bench-to-field transfer rate of abiotic stress-tolerant crops, as compared to crops designed to tolerate biotic stress. These differences underscore the complexity of abiotic stress-response mechanisms. Here, we suggest that breeding programs favoring yield-related quantitative physiological traits (QPTs; e.g., photosynthesis rate or stomatal conductance) have canalized those QPTs at their highest levels. This has affected the sensitivity of those QPTs to changing environmental conditions and those traits have become less plastic. We also suggest that breeding pressure has had an asymmetric impact on different QPTs, depending on their sensitivity to environmental conditions and their interactions with other QPTs. We demonstrate this asymmetric impact on the regulation of whole-plant water balance, showing how plastic membrane water content, stomatal conductance and leaf hydraulic conductance interact to canalize whole-organ water content. We suggest that a QPT’s plasticity is itself an important trait and that understanding this plasticity may help us to develop yield-optimized crops.

## Introduction

Unpredictable biotic and climatic (abiotic) factors have significant effects on crop production. It has been widely suggested that increasing crop yield is the most sustainable way toward the goal of global food security by 2050. To meet the demands of our increasing global population, crop yields need to double by 2050 [as reviewed by Ray et al., 2013]. Based on 1998–2008 yield trends of the top four global crops (i.e., maize, rice, wheat, and soybean), which constitute about two–thirds of current harvested global crop calories, it is estimated that the current rate of increase in crop yields is far slower than the ∼2.4% per year required to double the global crop yields by 2050 (Ray et al., 2013). Lobell et al. (2011) estimated the global impact of temperature and precipitation trends from 1980 to 2008 on average yield of the top four global crops and found that yields declined for each of those crops over that period, with the largest decline (5.5%) seen in wheat production. Due to the negative effects of abiotic environmental stresses such as drought, temperature extremes, poor soil quality and flooding, commercially grown crops achieve an average of only about 50% of their potential yield under field conditions (Hatfield and Walthall, 2015; Foyer et al., 2016). In contrast, biotic stresses such as insect pests contribute to a yield gap of approximately 10% (Kerchev et al., 2012), which rises to 50–80% in the absence of control measures (Bruce, 2010; Foyer et al., 2016), suggesting that abiotic stress can more sharply limit potential yield than biotic stress. Biotic-stress problems, which have much greater damage potential, are considered relatively straightforward to solve using resistant plants, as well as variety of other methods, including pesticides (Foyer et al., 2016).

## The Gap Between Basic Research and Abiotic Stress-Tolerant Crops

**FIGURE 1:**
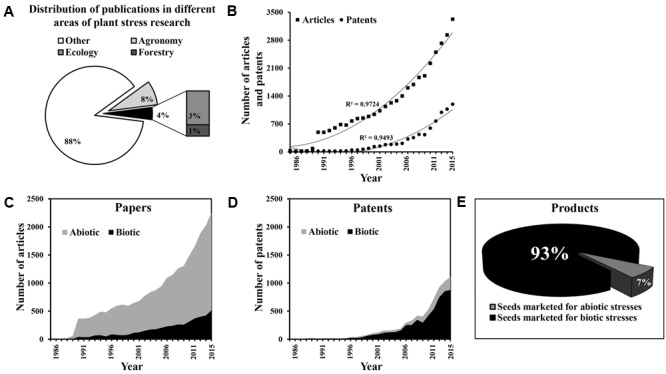
Translation of knowledge into practice: the papers-to-products ratio. **(A)** The relative proportion of articles published over three decades (1986 through 2015) in different areas of “plant stress” (biotic and abiotic) research (i.e., agronomy, ecology, forestry, and other). **(B)** Annual rate of articles published and patents filed in the fields of plant abiotic and biotic stress research. **(C)** Annual publication rate of articles concerning research into biotic and abiotic stress. **(D)** Annual filing rate of patents concerning research into biotic and abiotic stress. **(E)** The relative proportion of commercial seeds marketed as tolerant of biotic and abiotic stresses (as of January 2017).

In order to quantify and compare the complexity of abiotic and biotic stress responses, we categorized different types of stress in an *in silico* study and analyzed the gap between the number of research articles published and the actual release of stress-tolerant crops in the market, for each type of stress. We followed (Graff et al., 2013) with some modifications and used Web of Science Collection and Patent Search from Thomson Innovation^1^ (Supplementary Figures S1, S2) for the period 1986–2015. Under the category of “plant sciences” (consisting of 36 different areas), we found 34,757 published articles using the search term “plant stress,” of which 2918 articles (8%) were published in the area of agronomy, which represents crop ecosystems, and 1324 articles (4%) were published in the area of ecology and forestry, which represent natural ecosystems (**Figure 1A**). The number of articles published in the “plant stress” research area accounted for only ∼17% of overall “plant” biology research (Supplementary Figure S3A). During the same time period, 7659 DWPI (Derwent World Patents Index) families were filed in the area of “plant stress” research and we found that an increasing number of research papers were published and patents were filed over this period (**Figure 1B**). To distinguish between the total numbers of articles concerning biotic vs. abiotic stress, we chose query terms that we thought to be unique to each type of stress (Supplementary Figure S1). Our search revealed that within the general area of plant sciences, the number of publications on abiotic stress (23,883 articles) was more than five times the number of publications on biotic stress (4582 articles; **Figure 1C**). This ratio remained the same when we repeated the search among agronomy papers, but was 20 times greater in the field of ecology and 60 times greater in the field of forestry (Supplementary Figures S3B–E).

However, similar numbers of patents were found relating to abiotic (7050 DWPI families) and biotic stress (5496 DWPI families; **Figure 1D**). Moreover, the translation of these patents into products as reflected in marketed commercial seeds was very much tilted towards resistance/tolerance for biotic stress. We selected the top seven companies who filed patents related to “plant stress” research (i.e., Monsanto, DuPont, Syngenta, Bayer CropScience, Limagrain, Dow AgroSciences and BASF Plant Science) and thoroughly searched their websites (Supplementary Figure S4). Note that the published research papers and patents were collected from both public and private sectors while the commercially released varieties were taken into account only from the private sectors. Released (marketed) rice cultivars has not been particularly considered in a major way in this study simply because there is hardly any hybrid variety of rice marketed by the major seed companies. Yet, the fact remains that rice varieties have been generated and disseminated for many traits through research and development in the public systems (e.g., IRRI^2^), to the extent that it had major impact on the economies of developing countries such as Vietnam, Thailand and India. We found that cultivars exhibiting resistance/tolerance to different types of biotic stress accounted for ∼93% of the overall seed market (**Figure 1E**).

In our meta-analysis, the low bench-to-field transfer rate (ratio of patents to marketed commercial seeds) of abiotic stress-resistant crops as compared to crops designed to tolerate biotic stress emphasizes the complexity of abiotic stress responses. According to Moshelion and Altman (2015), one of the major bottlenecks in the development of plants that are resistant to or tolerant of abiotic stress is the lack of phenotyping tools to enable the translation of the massive quantity of research data into practical innovation in the field. The huge gap between basic research in plant responses to abiotic stress and the practical development of abiotic stress-tolerant crops (Graff et al., 2013) points to the complexity of the latter.

## The Complexity of Quantitative Physiological Traits Plasticity

**FIGURE 2:**
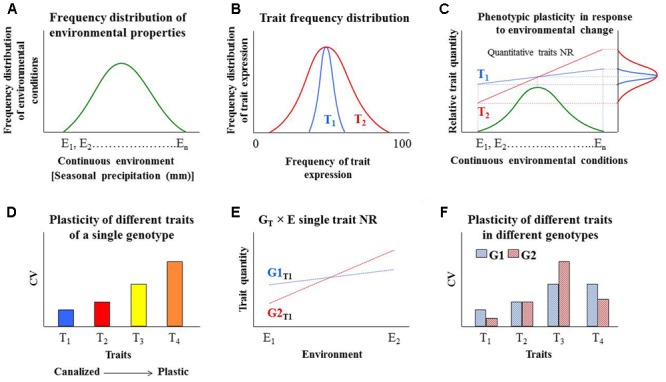
Hypothetical response patterns of multiple quantitative traits to different environmental conditions, within and between genotypes. **(A)** A normal frequency distribution of environmental conditions (E_1_, E_2_, ….. E_n_) in the habitat of a specific genotype. **(B)** The normal frequency distribution of two different quantitative traits, T_1_ and T_2_, of one genotype under the same environmental conditions presented in **(A)**. T_1_ is a more canalized trait and T_2_ is more plastic. These traits could also be presented in terms of **(C)** the traits’ norm of reaction (NR) with respect to the distribution of environmental conditions (i.e., T × E interactions). Each of these NRs and the plasticity of other traits of this genotype (or organism) could also be represented in a frequency distribution that could be represented as **(D)** standardized comparison as the coefficient of variation (CV), relatively scaled (T_1_, T_2_, T_3_ and T_4_). The lower the CV value, the more stable the trait. **(E)** Comparison of a specific trait across two genotypes (G1_T1_ and G2_T1_) when only two sets of environmental conditions are considered: stress conditions (E_1_) and optimal conditions (E_2_). **(F)** Standardized comparison of the relative plasticity of several traits within and between genotypes.

Plants encounter dynamic environmental conditions throughout their life cycles, which results in the expression of more than one phenotype, a phenomenon known as phenotypic plasticity (PP; i.e., the production of more than one phenotype from the same genotype in different environments; (Kooke et al., 2015; Davière and Achard, 2016; Ibañez et al., 2017). Phenotype is determined by genotype (G) and environmental factors (E) and PP is considered to be an evolutionary adaptation mechanism to changing and uncertain environmental conditions. The G × E interaction can be described by the linear model: P = G + E + G × E (Mackay, 2001; Bernardo, 2008; Wray and Visscher, 2008). Under changing environmental conditions, we would expect to see many examples of trait plasticity both within and between genotypes (see **Figure 2** for a schematic explanation of our hypothesis). Understanding PP is important for predicting changes in species distribution, community composition and crop productivity under changing environmental conditions (reviewed by Gratani, 2014). The stability of an environment (**Figure 2A**) may select for various combinations of phenotypic traits and with different levels of plasticity (Shemesh et al., 2010). The variety of traits represented by a single genotype (i.e., physiological, morphological, biochemical) represents a degree of phenotypic flexibility in which some traits are less stable (more plastic; e.g., number of flowers) under all physiological and environmental conditions, while other traits are more stable (canalized; e.g., flower structure and shape; **Figure 2B**).

Typically, the unique response pattern of a trait across two genotypes and within a given range of environmental conditions is referred to as that trait’s norm of reaction (NR; (El-Soda et al., 2014). The use of NR values allows the simple and rapid comparison of PP across a range of genotypes and environments. The degree of PP can be a crucial determinant of plant responses over both the short and long term (Nicotra et al., 2010). When climate conditions are changing rapidly, PP confers an adaptive advantage to the plant, allowing optimal resource acquisition or maximizing fitness in other ways (Pham and McConnaughay, 2014). We suggest that different (multiple) traits’ PP of the same organism could be compared based on their qualitative NR (**Figure 2C**). However, the PP trade-off costs are not clear.

The cost of plasticity is expressed as the reduction in the fitness of a genotype due to its phenotypic plasticity, as compared to fixed patterns of development that maintain homeostasis under stable conditions (Van Tienderen, 1991; DeWitt et al., 1998; Van Kleunen and Fischer, 2005, 2007). For example, if two genotypes, one with a plastic trait (e.g., height) and another with a canalized version of that same trait happen to grow in the same favorable environment, then the plastic genotype will be less fit than the canalized genotype due to the maintenance costs of the machinery required for plasticity (cost of plasticity; Van Kleunen and Fischer, 2005, 2007). Nevertheless, the trade-offs between plastic responses to different environmental stimuli and between plastic responses of different traits that share the same sensory and response pathways require particular attention and understanding those traits may also require a better understanding of the costs of plasticity (Van Kleunen and Fischer, 2005, 2007).

Yield-related physiological traits (e.g., stomatal conductance, photosynthesis, etc.) are generally quantitative traits (i.e., they depend on the cumulative actions of many genes and the environment). The relationship between yield-related quantitative physiological traits (QPTs) and the environment, namely QPT plasticity, is highly complex. We suggest that a better understanding of QPT plasticity may contribute to the development of crops with better stress-response optimization processes. Moreover, the variable sensitivity of different QPTs to the same environment results in a different response pattern for each QPT (i.e., QPT response sensitivity). Selection of the quantitative estimator of plasticity has an important impact on both the way plasticity is assessed and the ecological and evolutionary implications that can be mined (Valladares et al., 2006). Experimental as well as statistical methods can be used to study phenotypic variability (Geiler-Samerotte et al., 2013).

## Quantifying and Comparing Phenotypic Plasticity

Over the years, various statistical methods have been suggested for quantifying PP, including coefficient of variation (CV), the slope of norm of reaction, the Relative Distances Plasticity Index (RDPI), log transformation of the variance and the Box-Cox power transformation [reviewed by Valladares et al. (2006) and Pertoldi et al. (2014)]. Among those methods, CV has been widely used to measure PP, pending the trait’s normal distribution. Since CV is a standardized measure of frequency distribution and is unit-less, it enables the standardized comparison of the relative plasticity of several traits within a genotype (Wolfe and Mazer, 2005) **Figures 2C,D**. The utility of CV has a few limitations. First, it cannot be used to compare PP between different species (Valladares et al., 2006), but it can be used to compare different cultivars of the same species. Second, the CV of a composite measure is always less than the weighted average of the CVs of its parts (Bader and Hall, 1960; Pertoldi et al., 2014). Nevertheless, taking into consideration its limitations, CV is still considered to be one of the easiest standardized statistical tools for exploring phenotypic variability, in general, including developmental instability within a species. In fact, CV has been used as to compare the PP of a range of traits related to growth and morphology traits in Arabidopsis, barley, corn, tomato, oak and poplar under favorable and stress conditions (Volis et al., 1998; Valladares et al., 2002; Pliura et al., 2007; Dong et al., 2008; Gaudin et al., 2011; Kooke et al., 2015). We suggest that, since lower CV values are indicative of greater canalization (Toubiana et al., 2012) and higher CV values indicate greater plasticity (Fridman, 2015), CV can be calculated as a trait value and can serve as a tool for the standardized comparison of traits’ NR values simultaneously under varying environmental conditions (e.g., **Figure 2D**). Accordingly, we suggest that the typical NR for comparison of a specific trait across two (or more) genotypes (within the same species) will be clearly marked (e.g., G1_T1_ and G2_T1_; **Figure 2E**), in order to separate it from the standardized comparison of the relative plasticity of several traits within genotypes (**Figure 2F**).

## Physiological Trait Plasticity Hierarchy

Physiological trait plasticity is important for plant adaptation to adverse environments where morphological and anatomical plasticity play less dominant roles (Zunzunegui et al., 2009; Gratani, 2014). Like other quantitative traits, QPTs show different degrees of sensitivity to the same environmental signals. For example, earlier studies have shown that, under stressful conditions, leaf hydraulic conductance (K_leaf_) decreases more quickly than the stomatal conductance (g_s_) of the same leaf (Sack and Scoffoni, 2012), suggesting that K_leaf_ in more sensitive to reductions in leaf water potential (ψ) than g_s_ is. Additional evidence for QPT sensitivity differences was provided by Martre et al. (2002), who showed that exposing a whole plant to drought stress resulted in differential QPT responses. In that work, whole-plant hydraulic conductance was found to be twice as sensitive as the transpiration rate to declining soil water content (reaching 50% of its maximum value 2 days before the transpiration of the same plants reached 50% of their maximum value) and these two QPTs were more sensitive to drought than ψ and osmotic or turgor pressure, which remained unaffected by drought stress for several more days (Martre et al., 2002). Also, the circadian plasticity of root hydraulic conductance was found to contribute to the acclimation to water stress by increasing the root water uptake, thereby favoring growth and photosynthesis (Caldeira et al., 2014). Furthermore, Topp (2016) commented that the most yield stable genotypes have the most plastic root traits (growth and architecture) among environments.

A meta-analysis comparing various plant water-relation QPTs within and across species across biomes revealed that relative water content (RWC) exhibits a very narrow range of variation, as compared to other QPTs (Bartlett et al., 2012). Moreover, Sade et al. (2012) showed that RWC is a more stable trait than leaf water potential, in the context of characterizing isohydric plant behavior. This canalization of the RWC trait (i.e., maintaining of critical water level of the plant) may be related to its importance for evolutionary fitness (i.e., seed development under uncertain environmental conditions) and productivity (i.e., maximizing crop yield).

One possible explanation for this QPT hierarchy may be related to the regulation of leaf steady-state water status. For example, Simonin et al. (2014) suggested that the positive dependence of dynamic leaf hydraulic conductance on transpiration tends to minimize or reduce water potential gradients along the soil–plant–atmosphere continuum, thereby stabilizing leaf water content (LWC; Simonin et al., 2014). This is due to the fact that stomatal conductance depends on leaf water potential, soil water potential, the movement of water through the soil and plant, and xylem hydraulic resistance (Comstock and Mencuccini, 1998; Tuzet et al., 2003; Klein, 2014; Dry and Loveys, 2015; Feng et al., 2017). These results suggest that some key QPTs of a genotype exhibit different sensitivity thresholds under the same environmental conditions.

Earlier studies have shown hierarchy in the elasticities of the productive traits of seed number and size and proposed that the elasticities of related traits may be negatively related to one another within a hierarchy (Bradshaw, 1965; Sadras, 2007; Sadras et al., 2009). Accordingly, we suggest that some interactions between plant-water-regulation QPTs indicate the existence of a hierarchical regulatory mechanism that is transduced down from RWC to the molecular level of aquaporin activity. That is, the stable RWC of the plant is a result of regulation of g_s_ (leaf water outflow) and K_leaf_ (leaf water inflow). Nonetheless, g_s_ and K_leaf_ are controlled by the activity of the specialized cells (guard cells and bundle sheath cells, respectively) and their membrane osmotic water permeability (P_f_), in particular (Shatil-Cohen et al., 2011). P_f_ is known to be regulated by aquaporin activity (reviewed by Yaaran and Moshelion, 2016). Aquaporins are activated by several transcriptional and post-translational mechanisms namely, phosphorylation (Johansson et al., 1998; Guenther et al., 2003), heteromerization (Fetter et al., 2004), pH (Tournaire-Roux et al., 2003), Ca^2+^ (Gerbeau et al., 2002), pressure (Zhu et al., 2002, 2004) and solute gradients (Ye et al., 2004). Regulation of aquaporin trafficking (through insertion into/or removal from the membrane) may also represent a way to modulate membrane water permeability, as has been demonstrated in mammals (reviewed by Brown et al., 1998; Brown, 2003). This concept is supported in plants by the non-uniform subcellular localization of aquaporins, for example, among membrane domains (reviewed by Maurel et al., 2009), or in response to abiotic stress, which caused trafficking of TIP from the tonoplast to the cytosol (e.g., Vera-Estrella et al., 2004). We suggest that this complex aquaporin function contributes to high plasticity of the cellular osmotic water permeability (P_f_) regulation (Kaldenhoff et al., 1998; Shatil-Cohen et al., 2011, 2014), which further modulates ψ and K_leaf_ (Martre et al., 2002; Prado and Maurel, 2013).

**FIGURE 3:**
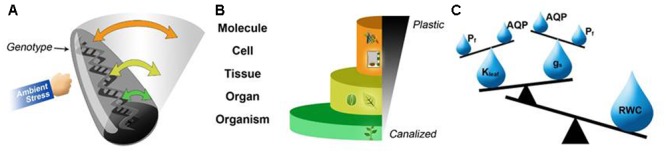
Our model of the hierarchical plasticity of QPTs and the risk-intensifying canalization that has resulted from breeding pressure. **(A)** The Roly-Poly model represents the response magnitudes of the different QPTs (plasticity, represented by the swing amplitude) of a genotype (the Roly-Poly) exposed to environmental stress (punching hand). The cellular level, which is the most sensitive to changes in the environment, is characterized by a large variety of regulatory mechanisms, which lead to high levels of plasticity. On the other hand, the organism-level QPTs are the most canalized, which results in minimal changes in their values (i.e., low plasticity). **(B)** This molecular-to-organ hierarchical regulation relationship enables the high plasticity of reactions involving molecular/cellular traits and helps to maintain the stability of higher-order traits. For example, for the regulation of plant water relations: **(C)** the hierarchy of water-balance-related QPTs in which the stability of RWC, a core QPT that is essential for plant survival and productivity, is achieved by balancing tissue-related QPTs (K_leaf_ and g_s_), which, in turn, are maintained by the balance between cellular-level QPTs, namely aquaporin (AQP) regulation and the P_f_ properties of guard cells and bundle-sheath cells.

We tried to simplify the complex interactions and varying sensitivity of these QPTs in a schematic diagram (**Figure 3**). The Roly-Poly model (**Figure 3A**) represents the degree of response of different QPTs of a genotype exposed to environmental stresses. When a genome is under stress (represented as the punched Roly-Poly), not all of the genes are affected similarly (top to bottom along the vertical axis). Some exhibit greater differences in their expression patterns (i.e., exhibit more plastic behavior), while others remain stable (analogous to the base of the Roly-Poly). Accordingly, the variable plasticity of the expression patterns of different genes is translated into variable expression, activity and interaction of the respective proteins which in turn determines the cellular, organelle and whole plant NRs of different QPTs. QPTs can be ranked based on their level of plasticity and grouped from lower (e.g., molecules, cells, tissues, etc.) to higher levels, namely organs and organisms. Arrangement of the QPTs from lower to higher levels of organization (half pyramid scheme) suggests that more stable QPTs are found at the organism level, more plastic QPTs are seen at the tissue and cellular levels and that the most plastic QPTs are found at the molecular level (pyramid base, middle and top respectively; **Figure 3B**). Therefore, going back to our previous example, we suggest that the highly responsive nature of cellular osmotic permeability (via aquaporin expression and activity) helps to maintain more stable K_leaf_ and g_s_, which together maintain a stable leaf water status (RWC and ψ_leaf_, **Figure 3C**). Our QPT regulation hierarchy model suggests a supportive complimentary functional approach to the theory presented by Sadras and Richards (2014), which states that a trait scales up from lower (e.g., molecule, cell, tissue, etc.) to higher levels (e.g., individual, population, community, etc.) of organization, if it remains agronomically relevant at higher levels and is eventually expressed at the population level, at which yield is defined. In addition, characterization of dynamic environments and understanding their stress gradients is also important in the understanding of plastic response.

## Putative Implications for Breeding

**FIGURE 4:**
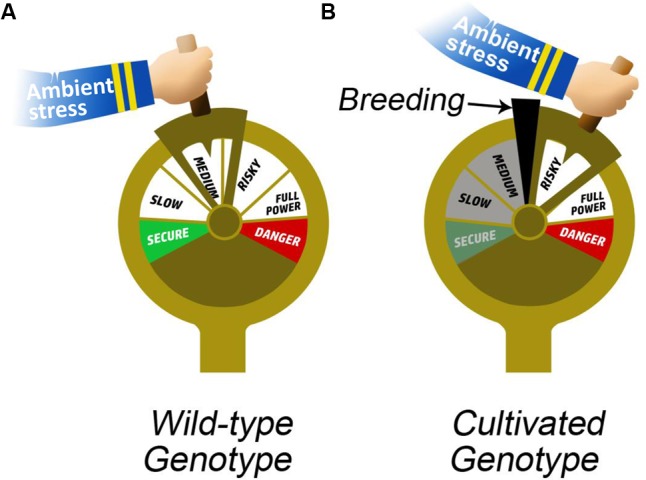
Impact of breeding on physiological plasticity. The hand holding the boat throttle represents ambient stress. **(A)** In the case of a wild variety, the throttle is capable of moving freely both ways, representing flexibility (the plasticity of the QPTs). In contrast, in the case of a cultivated variety, **(B)** the wedge in the throttle represents breeding pressure and allows the throttle to move only at the risky end (canalization of QPTs), forcing the plant to “perform or die.”

Crop breeding programs have traditionally aimed to increase productivity, which has had the side effect of increasing absolute values of key yield-related QPTs that are important for the regulation of plant water balance [e.g., stomatal conductance (Richards et al., 2010), leaf hydraulic conductance (Sack and Holbrook, 2006) and photosynthesis (Takai et al., 2013)]. Selective breeding is often conducted under a single set of environmental conditions (Nicotra et al., 2010), therefore, the breeding pressure has had an asymmetric impact on different QPTs, depending on their sensitivity to environmental conditions and their interactions with other QPTs. These dynamic QPTs play key roles in the plant water-balance regulation (analogy to an engine with many degrees of freedom; **Figure 4A**) that underlies plant–environment interactions. The increased water use of crop plants (due to increasing absolute values of g_s_ and K_leaf_ QPTs – breeding by-products) is accompanied by reduced plasticity of those traits (Nicotra et al., 2010). The canalization of these traits at their higher levels may be one of the key factors in increasing the susceptibility of crops to environmental stresses (analogy to an engine stuck in high gear; **Figure 4B**). Therefore, we suggest that while breeding programs have led to significant improvements in yield under optimal and targeted stress conditions, they have also led to increased susceptibility to various drought and suboptimal environmental conditions, as a result of reduced plasticity, that is, there has been a trade-off between productivity and survivability. This situation may also be responsible for the fact that it so hard to re-breed highly productive cultivars to be tolerant of abiotic stress (**Figure 1**). An ideal pattern of plant behavior (ideotype) depends on agronomic needs and the level of environment-related risk, is dynamic and will result in maximum crop yield in a given environmental scenario (Negin and Moshelion, 2017).

## Conclusion

We suggest that the high degree of plasticity of QPTs in wild-type plants might be part of their strategy for survival in an unstable environment. Traditional breeding has increased the absolute values of these traits and made them more canalized, thereby making the plants less responsive to changing environmental conditions. This trade-off between plasticity and productivity limits our ability to improve crop tolerance of certain types of abiotic stress. We suggest that, the breeding programs may consider increasing the plasticity level of yield-related QPTs as a mean to improve crop stress performance. Nevertheless, a better understanding of the mechanism controlling QPT stability would enable us to develop cultivars that exhibit improved yield-optimizing behavior. However, that behavior is likely to be beneficial only under certain conditions and different plant behaviors should be selected for cultivation in different environments.

## Author Contributions

All authors have contributed substantially in conceiving the idea, and designing and interpretation of the work. All authors have made significant contribution to the preparation and revision of drafts, and have given final approval for publication.

## Conflict of Interest Statement

The authors declare that the research was conducted in the absence of any commercial or financial relationships that could be construed as a potential conflict of interest.

## Footnotes

1 http://info.thomsoninnovation.com

2 https://sites.google.com/a/irri.org/released-rice-varieties/released-varieties/2014

## References

[B1] Bader R. S. Hall J. S. (1960). Osteometric variation and function in bats. *Evolution* 14 8–17. 10.1111/j.1558-5646.1960.tb03052.x

[B2] Bartlett M. K. Scoffoni C. Sack L. (2012). The determinants of leaf turgor loss point and prediction of drought tolerance of species and biomes: a global meta-analysis. *Ecol. Lett.* 15 393–405. 10.1111/j.1461-0248.2012.01751.x 22435987

[B3] Bernardo R. (2008). Molecular markers and selection for complex traits in plants: learning from the last 20 years. *Crop Sci.* 48 1649–1664. 10.2135/cropsci2008.03.0131

[B4] Bradshaw A. D. (1965). Evolutionary significance of phenotypic plasticity in plants. *Adv. Genet.* 13 115–155. 10.1016/S0065-2660(08)60048-6

[B5] Brown D. (2003). The ins and outs of aquaporin-2 trafficking. *Am. J. Physiol. Renal Physiol.* 284 F893–F901. 10.1152/ajprenal.00387.2002 12676734

[B6] Brown D. Katsura T. Gustafson C. E. (1998). Cellular mechanisms of aquaporin trafficking. *Am. J. Physiol. Renal Physiol.* 275 F328–F331. 10.1152/ajprenal.1998.275.3.F328 9729503

[B7] Bruce T. J. (2010). Tackling the threat to food security caused by crop pests in the new millennium. *Food Sec.* 2 133–141. 10.1007/s12571-010-0061-8

[B8] Caldeira C. F. Jeanguenin L. Chaumont F. Tardieu F. (2014). Circadian rhythms of hydraulic conductance and growth are enhanced by drought and improve plant performance. *Nat. Commun.* 5:5365. 10.1038/ncomms6365 25370944 PMC4241992

[B9] Comstock J. Mencuccini M. (1998). Control of stomatal conductance by leaf water potential in *Hymenoclea salsola* (T. & G.), a desert subshrub. *Plant Cell Environ.* 21 1029–1038. 10.1046/j.1365-3040.1998.00353.x

[B10] Davière J.-M. Achard P. (2016). A pivotal role of DELLAs in regulating multiple hormone signals. *Mol. Plant* 9 10–20. 10.1016/j.molp.2015.09.011 26415696

[B11] DeWitt T. J. Sih A. Wilson D. S. (1998). Costs and limits of phenotypic plasticity. *Trends Ecol. Evol.* 13 77–81. 10.1016/S0169-5347(97)01274-3 21238209

[B12] Dong Q. Louarn G. Wang Y. Barczi J.-F. de Reffye P. (2008). Does the structure–function model GREENLAB deal with crop phenotypic plasticity induced by plant spacing? A case study on tomato. *Ann. Bot.* 101 1195–1206. 10.1093/aob/mcm317 18199575 PMC2710282

[B13] Dry P. Loveys B. (2015). Grapevine shoot growth and stomatal conductance are reduced when part of the root system is dried. *Vitis* 38 151–156.

[B14] El-Soda M. Malosetti M. Zwaan B. J. Koornneef M. Aarts M. G. (2014). Genotype × environment interaction QTL mapping in plants: lessons from *Arabidopsis*. *Trends Plant Sci.* 19 390–398. 10.1016/j.tplants.2014.01.001 24491827

[B15] Feng X. Dawson T. E. Ackerly D. D. Santiago L. S. Thompson S. E. (2017). Reconciling seasonal hydraulic risk and plant water use through probabilistic soil–plant dynamics. *Glob. Change Biol.* 23 3758–3769. 10.1111/gcb.13640 28132414

[B16] Fetter K. Van Wilder V. Moshelion M. Chaumont F. (2004). Interactions between plasma membrane aquaporins modulate their water channel activity. *Plant Cell* 16 215–228. 10.1105/tpc.017194 14671024 PMC301406

[B17] Foyer C. H. Rasool B. Davey J. W. Hancock R. D. (2016). Cross-tolerance to biotic and abiotic stresses in plants: a focus on resistance to aphid infestation. *J. Exp. Bot.* 67 2025–2037. 10.1093/jxb/erw079 26936830

[B18] Fridman E. (2015). Consequences of hybridization and heterozygosity on plant vigor and phenotypic stability. *Plant Sci.* 232 35–40. 10.1016/j.plantsci.2014.11.014 25617321

[B19] Gaudin A. C. M. McClymont S. A. Holmes B. M. Lyons E. Raizada M. N. (2011). Novel temporal, fine-scale and growth variation phenotypes in roots of adult-stage maize (*Zea mays* L.) in response to low nitrogen stress. *Plant Cell Environ.* 34 2122–2137. 10.1111/j.1365-3040.2011.02409.x 21848860

[B20] Geiler-Samerotte K. Bauer C. Li S. Ziv N. Gresham D. Siegal M. (2013). The details in the distributions: why and how to study phenotypic variability. *Curr. Opin. Biotechnol.* 24 752–759. 10.1016/j.copbio.2013.03.010 23566377 PMC3732567

[B21] Gerbeau P. Amodeo G. Henzler T. Santoni V. Ripoche P. Maurel C. (2002). The water permeability of *Arabidopsis* plasma membrane is regulated by divalent cations and pH. *Plant J.* 30 71–81. 10.1046/j.1365-313X.2002.01268.x 11967094

[B22] Graff G. Hochman G. Zilberman D. (2013). “The research, development, commercialization, and adoption of drought and stress-tolerant crops,” in *Crop Improvement under Adverse Conditions* eds Tuteja N. Gill S. S. (New York, NY: Springer) 1–33. 10.1007/978-1-4614-4633-0_1

[B23] Gratani L. (2014). Plant phenotypic plasticity in response to environmental factors. *Adv. Bot.* 2014:208747 10.1155/2014/208747

[B24] Guenther J. F. Chanmanivone N. Galetovic M. P. Wallace I. S. Cobb J. A. Roberts D. M. (2003). Phosphorylation of soybean nodulin 26 on serine 262 enhances water permeability and is regulated developmentally and by osmotic signals. *Plant Cell* 15 981–991. 10.1105/tpc.009787 12671092 PMC152343

[B25] Hatfield J. L. Walthall C. L. (2015). Meeting global food needs: realizing the potential via genetics × environment × management interactions. *Agron. J.* 107 1215–1226. 10.2134/agronj15.0076

[B26] Ibañez C. Poeschl Y. Peterson T. Bellstädt J. Denk K. Gogol-Döring A. (2017). Ambient temperature and genotype differentially affect developmental and phenotypic plasticity in *Arabidopsis thaliana*. *BMC Plant Biol.* 17:114. 10.1186/s12870-017-1068-5 28683779 PMC5501000

[B27] Johansson I. Karlsson M. Shukla V. K. Chrispeels M. J. Larsson C. Kjellbom P. (1998). Water transport activity of the plasma membrane aquaporin PM28A is regulated by phosphorylation. *Plant Cell* 10 451–459. 10.1105/tpc.10.3.451 9501117 PMC144000

[B28] Kaldenhoff R. Grote K. Zhu J. J. Zimmermann U. (1998). Significance of plasmalemma aquaporins for water-transport in *Arabidopsis thaliana*. *Plant J.* 14 121–128. 10.1046/j.1365-313X.1998.00111.x 9681029

[B29] Kerchev P. I. Fenton B. Foyer C. H. Hancock R. D. (2012). Plant responses to insect herbivory: interactions between photosynthesis, reactive oxygen species and hormonal signalling pathways. *Plant Cell Environ.* 35 441–453. 10.1111/j.1365-3040.2011.02399.x 21752032

[B30] Klein T. (2014). The variability of stomatal sensitivity to leaf water potential across tree species indicates a continuum between isohydric and anisohydric behaviours. *Funct. Ecol.* 28 1313–1320. 10.1111/1365-2435.12289

[B31] Kooke R. Johannes F. Wardenaar R. Becker F. Etcheverry M. Colot V. (2015). Epigenetic basis of morphological variation and phenotypic plasticity in *Arabidopsis thaliana*. *Plant Cell* 27 337–348. 10.1105/tpc.114.133025 25670769 PMC4456930

[B32] Lobell D. B. Schlenker W. Costa-Roberts J. (2011). Climate trends and global crop production since 1980. *Science* 333 616–620. 10.1126/science.1204531 21551030

[B33] Mackay T. F. C. (2001). The genetic architecture of quantitative traits. *Annu. Rev. Genet.* 35 303–339. 10.1146/annurev.genet.35.102401.090633 11700286

[B34] Martre P. Morillon R. Barrieu F. North G. B. Nobel P. S. Chrispeels M. J. (2002). Plasma membrane aquaporins play a significant role during recovery from water deficit. *Plant Physiol.* 130 2101–2110. 10.1104/pp.009019 12481094 PMC166722

[B35] Maurel C. Santoni V. Luu D. T. Wudick M. M. Verdoucq L. (2009). The cellular dynamics of plant aquaporin expression and functions. *Curr. Opin. Plant Biol.* 12 690–698. 10.1016/j.pbi.2009.09.002 19783200

[B36] Moshelion M. Altman A. (2015). Current challenges and future perspectives of plant and agricultural biotechnology. *Trends Biotechnol.* 33 337–342. 10.1016/j.tibtech.2015.03.001 25842169

[B37] Negin B. Moshelion M. (2017). The advantages of functional phenotyping in pre-field screening for drought-tolerant crops. *Funct. Plant Biol.* 44 107–118. 10.1071/FP16156 32480550

[B38] Nicotra A. B. Atkin O. K. Bonser S. P. Davidson A. M. Finnegan E. Mathesius U. (2010). Plant phenotypic plasticity in a changing climate. *Trends Plant Sci.* 15 684–692. 10.1016/j.tplants.2010.09.008 20970368

[B39] Pertoldi C. Bundgaard J. Loeschcke V. Barker J. S. F. (2014). The phenotypic variance gradient–a novel concept. *Ecol. Evol.* 4 4230–4236. 10.1002/ece3.1298 25540685 PMC4267862

[B40] Pham B. McConnaughay K. (2014). “Plant phenotypic expression in variable environments,” in *Ecology and the Environment* Vol. 8 ed. Monson R. K. (New York, NY: Springer) 119–141. 10.1007/978-1-4614-7501-9_16

[B41] Pliura A. Zhang S. MacKay J. Bousquet J. (2007). Genotypic variation in wood density and growth traits of poplar hybrids at four clonal trials. *For. Ecol. Manag.* 238 92–106. 10.1016/j.foreco.2006.09.082

[B42] Prado K. Maurel C. (2013). Regulation of leaf hydraulics: from molecular to whole plant levels. *Front. Plant Sci.* 4:255. 10.3389/fpls.2013.00255 23874349 PMC3711007

[B43] Ray D. K. Mueller N. D. West P. C. Foley J. A. (2013). Yield trends are insufficient to double global crop production by 2050. *PLOS ONE* 8:e66428. 10.1371/journal.pone.0066428 23840465 PMC3686737

[B44] Richards R. A. Rebetzke G. J. Watt M. Condon A. T. Spielmeyer W. Dolferus R. (2010). Breeding for improved water productivity in temperate cereals: phenotyping, quantitative trait loci, markers and the selection environment. *Funct. Plant Biol.* 37 85–97. 10.1071/FP09219

[B45] Sack L. Holbrook N. M. (2006). Leaf hydraulics. *Annu. Rev. Plant Biol.* 57 361–381. 10.1146/annurev.arplant.56.032604.144141 16669766

[B46] Sack L. Scoffoni C. (2012). Measurement of leaf hydraulic conductance and stomatal conductance and their responses to irradiance and dehydration using the evaporative flux method (EFM). *J. Vis. Exp.* 70:e4179. 10.3791/4179 23299126 PMC3577864

[B47] Sade N. Gebremedhin A. Moshelion M. (2012). Risk-taking plants: anisohydric behavior as a stress-resistance trait. *Plant Signal. Behav.* 7 767–770. 10.4161/psb.20505 22751307 PMC3583960

[B48] Sadras V. Reynolds M. De la Vega A. Petrie P. Robinson R. (2009). Phenotypic plasticity of yield and phenology in wheat, sunflower and grapevine. *Field Crops Res.* 110 242–250. 10.1016/j.fcr.2008.09.004

[B49] Sadras V. Richards R. (2014). Improvement of crop yield in dry environments: benchmarks, levels of organisation and the role of nitrogen. *J. Exp. Bot.* 65 1981–1995. 10.1093/jxb/eru061 24638898

[B50] Sadras V. O. (2007). Evolutionary aspects of the trade-off between seed size and number in crops. *Field Crops Res.* 100 125–138. 10.1016/j.fcr.2006.07.004

[B51] Shatil-Cohen A. Attia Z. Moshelion M. (2011). Bundle-sheath cell regulation of xylem- mesophyll water transport via aquaporins under drought stress: a target of xylem-borne ABA? *Plant J.* 67 72–80. 10.1111/j.1365-313X.2011.04576.x 21401747

[B52] Shatil-Cohen A. Sibony H. Draye X. Chaumont F. Moran N. Moshelion M. (2014). Measuring the osmotic water permeability coefficient (Pf) of spherical cells: isolated plant protoplasts as an example. *J. Vis. Exp.* 92:e51652. 10.3791/51652 25350534 PMC4841294

[B53] Shemesh H. Arbiv A. Gersani M. Ovadia O. Novoplansky A. (2010). The effects of nutrient dynamics on root patch choice. *PLOS ONE* 5:e10824. 10.1371/journal.pone.0010824 20520811 PMC2877079

[B54] Simonin K. A. Burns E. Choat B. Barbour M. M. Dawson T. E. Franks P. J. (2014). Increasing leaf hydraulic conductance with transpiration rate minimizes the water potential drawdown from stem to leaf. *J. Exp. Bot.* 66 1303–1315. 10.1093/jxb/eru481 25547915 PMC4339593

[B55] Takai T. Adachi S. Taguchi-Shiobara F. Sanoh-Arai Y. Iwasawa N. Yoshinaga S. (2013). A natural variant of NAL1, selected in high-yield rice breeding programs, pleiotropically increases photosynthesis rate. *Sci. Rep.* 3:2149. 10.1038/srep02149 23985993 PMC3756344

[B56] Topp C. N. (2016). Hope in change: the role of root plasticity in crop yield stability. *Plant Physiol.* 172 5–6. 10.1104/pp.16.01257 27578845 PMC5074644

[B57] Toubiana D. Semel Y. Tohge T. Beleggia R. Cattivelli L. Rosental L. (2012). Metabolic profiling of a mapping population exposes new insights in the regulation of seed metabolism and seed, fruit, and plant relations. *PLOS Genet.* 8:e1002612. 10.1371/journal.pgen.1002612 22479206 PMC3315483

[B58] Tournaire-Roux C. Sutka M. Javot H. Gout E. Gerbeau P. Luu D. T. (2003). Cytosolic pH regulates root water transport during anoxic stress through gating of aquaporins. *Nature* 425 393–397. 10.1038/nature01853 14508488

[B59] Tuzet A. Perrier A. Leuning R. (2003). A coupled model of stomatal conductance, photosynthesis and transpiration. *Plant Cell Environ.* 26 1097–1116. 10.1046/j.1365-3040.2003.01035.x

[B60] Valladares F. Balaguer L. Martinez-Ferri E. Perez-Corona E. Manrique E. (2002). Plasticity, instability and canalization: is the phenotypic variation in seedlings of sclerophyll oaks consistent with the environmental unpredictability of Mediterranean ecosystems? *New Phytol.* 156 457–467. 10.1046/j.1469-8137.2002.00525.x 33873566

[B61] Valladares F. Sanchez-Gomez D. Zavala M. A. (2006). Quantitative estimation of phenotypic plasticity: bridging the gap between the evolutionary concept and its ecological applications. *J. Ecol.* 94 1103–1116. 10.1111/j.1365-2745.2006.01176.x

[B62] Van Kleunen M. Fischer M. (2005). Constraints on the evolution of adaptive phenotypic plasticity in plants. *New Phytol.* 166 49–60. 10.1111/j.1469-8137.2004.01296.x 15760350

[B63] Van Kleunen M. Fischer M. (2007). Progress in the detection of costs of phenotypic plasticity in plants. *New Phytol.* 176 727–730. 10.1111/j.1469-8137.2007.02296.x 17997755

[B64] Van Tienderen P. H. (1991). Evolution of generalists and specialist in spatially heterogeneous environments. *Evolution* 45 1317–1331. 10.1111/j.1558-5646.1991.tb02638.x 28563821

[B65] Vera-Estrella R. Barkla B. J. Bohnert H. J. Pantoja O. (2004). Novel regulation of aquaporins during osmotic stress. *Plant Physiol.* 135 2318–2329. 10.1104/pp.104.044891 15299122 PMC520800

[B66] Volis S. Mendlinger S. Olsvig-Whittaker L. Safriel U. N. Orlovsky N. (1998). Phenotypic variation and stress resistance in core and peripheral populations of *Hordeum spontaneum*. *Biodivers. Conserv.* 7 799–813. 10.1023/A:1008844504010

[B67] Wolfe L. M. Mazer S. J. (2005). Patterns of phenotypic plasticity and their fitness consequences in wild radish (*Raphanus sativus*: Brassicaceae). *Int. J. Plant Sci.* 166 631–640. 10.1086/430194

[B68] Wray N. Visscher P. (2008). Estimating trait heritability. *Nat. Educ.* 1 29.

[B69] Yaaran A. Moshelion M. (2016). Role of aquaporins in a composite model of water transport in the leaf. *Int. J. Mol. Sci.* 17:1045. 10.3390/ijms17071045 27376277 PMC4964421

[B70] Ye Q. Wiera B. Steudle E. (2004). A cohesion/tension mechanism explains the gating of water channels (aquaporins) in *Chara* internodes by high concentration. *J. Exp. Bot.* 55 449–461. 10.1093/jxb/erh040 14739267

[B71] Zhu F. Tajkhorshid E. Schulten K. (2002). Pressure-induced water transport in membrane channels studied by molecular dynamics. *Biophys. J.* 83 154–160. 10.1016/S0006-3495(02)75157-6 12080108 PMC1302135

[B72] Zhu F. Tajkhorshid E. Schulten K. (2004). Theory and simulation of water permeation in aquaporin-1. *Biophys. J.* 86 50–57. 10.1016/S0006-3495(04)74082-5 14695248 PMC1303818

[B73] Zunzunegui M. Ain-Lhout F. Barradas M. D. Álvarez-Cansino L. Esquivias M. Novo F. G. (2009). Physiological, morphological and allocation plasticity of a semi-deciduous shrub. *Acta Oecol.* 35 370–379. 10.1016/j.actao.2009.02.004

