# Supplementary material for: To Produce or to Survive: How Plastic Is Your Crop Stress Physiology?
Source: Front Plant Sci. 2017 Dec 5;8:2067. doi: 10.3389/fpls.2017.02067 (PMC5723404; doi:10.3389/fpls.2017.02067)
Supplement: Supplementary file 1 [file Presentation_1.pptx]

## Slide 1
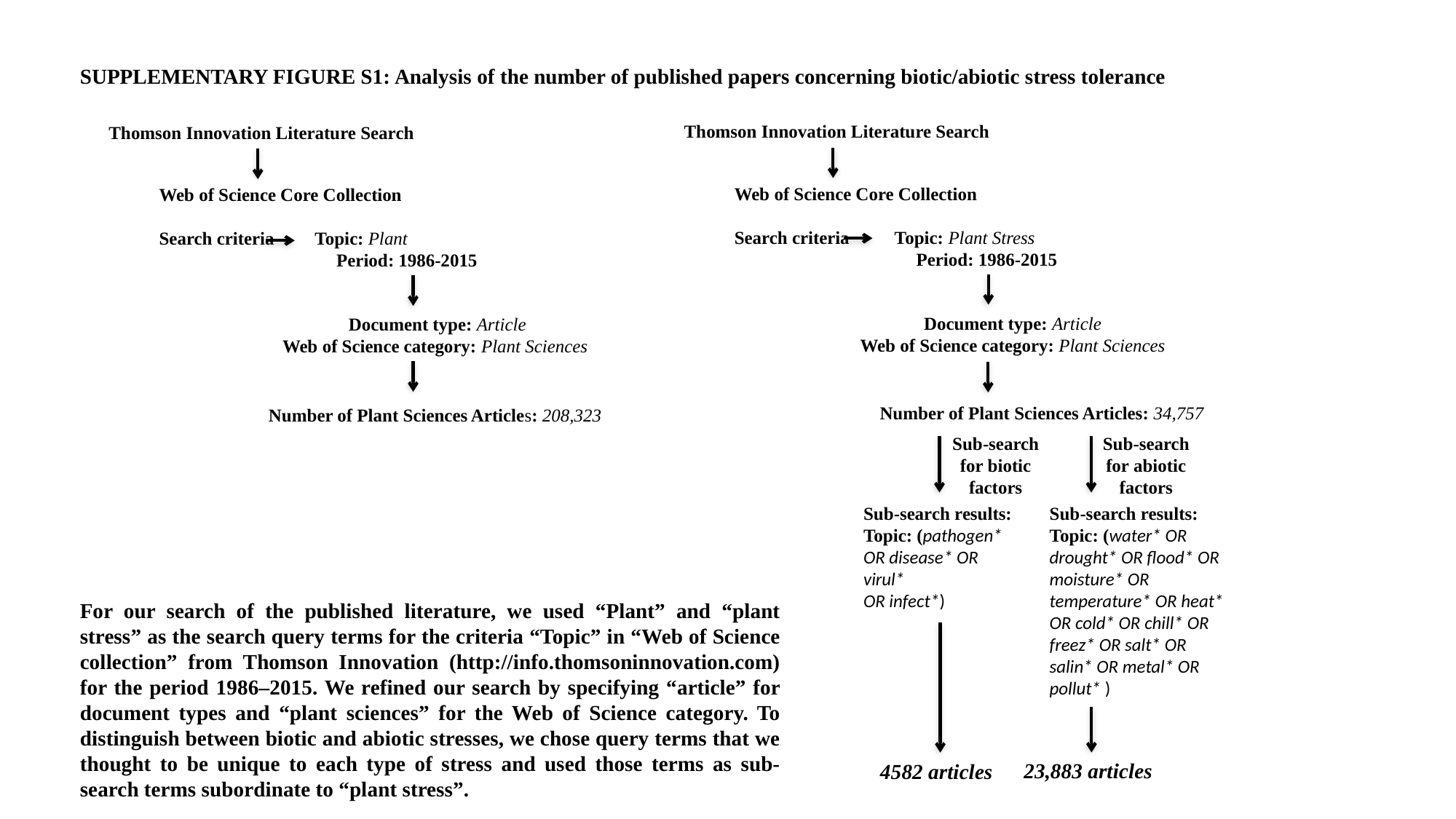

SUPPLEMENTARY FIGURE S1: Analysis of the number of published papers concerning biotic/abiotic stress tolerance
Thomson Innovation Literature Search
Thomson Innovation Literature Search
Web of Science Core Collection
Search criteria Topic: Plant Stress
 	 Period: 1986-2015
Web of Science Core Collection
Search criteria Topic: Plant
 	 Period: 1986-2015
Document type: Article
Web of Science category: Plant Sciences
Document type: Article
Web of Science category: Plant Sciences
Number of Plant Sciences Articles: 34,757
Number of Plant Sciences Articles: 208,323
Sub-search for biotic factors
Sub-search for abiotic factors
Sub-search results:
Topic: (water* OR drought* OR flood* OR moisture* OR temperature* OR heat* OR cold* OR chill* OR freez* OR salt* OR salin* OR metal* OR pollut* )
Sub-search results:
Topic: (pathogen* OR disease* OR virul*
OR infect*)
For our search of the published literature, we used “Plant” and “plant stress” as the search query terms for the criteria “Topic” in “Web of Science collection” from Thomson Innovation (http://info.thomsoninnovation.com) for the period 1986–2015. We refined our search by specifying “article” for document types and “plant sciences” for the Web of Science category. To distinguish between biotic and abiotic stresses, we chose query terms that we thought to be unique to each type of stress and used those terms as sub-search terms subordinate to “plant stress”.
23,883 articles
4582 articles

## Slide 2
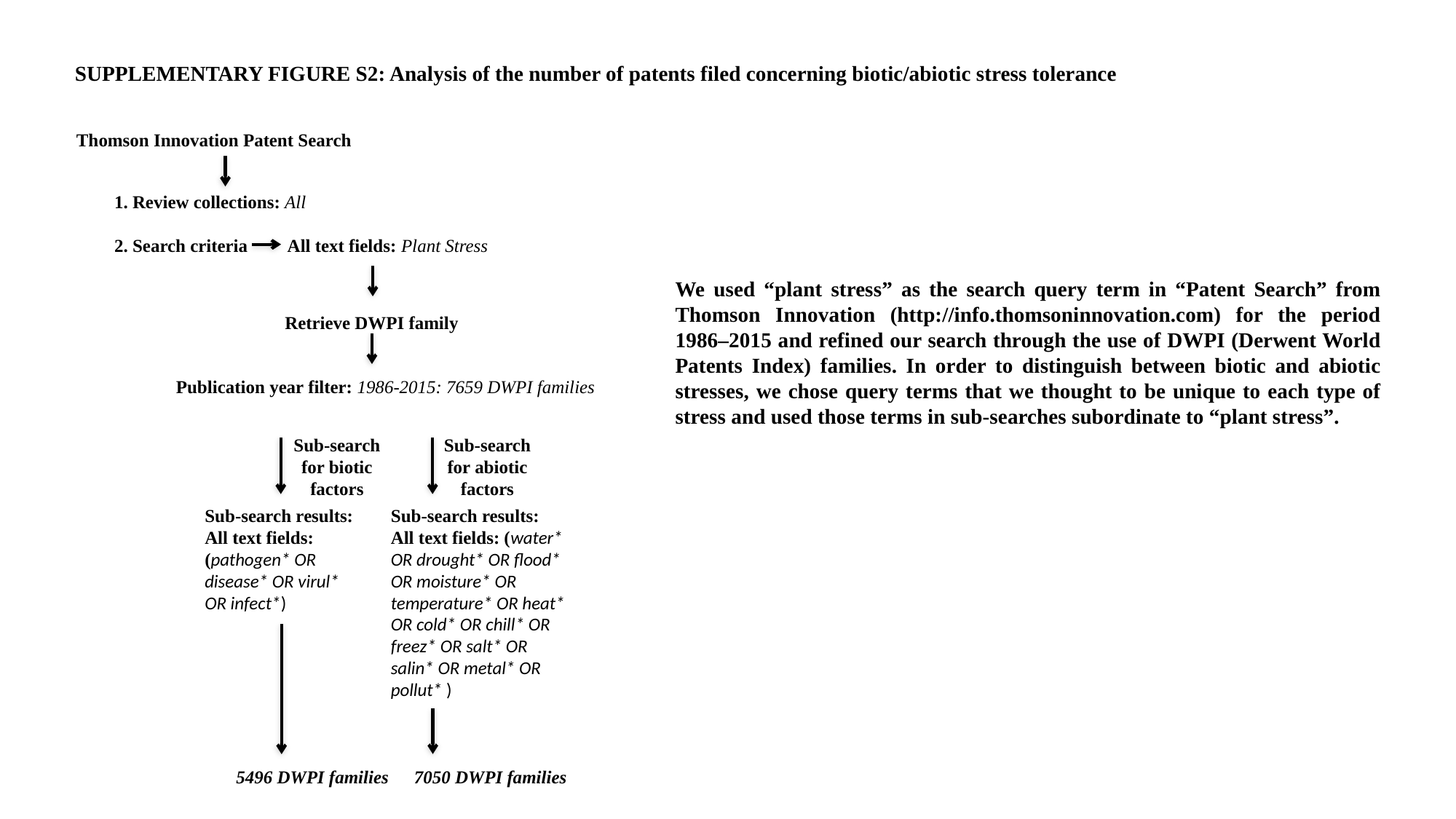

SUPPLEMENTARY FIGURE S2: Analysis of the number of patents filed concerning biotic/abiotic stress tolerance
Thomson Innovation Patent Search
 Review collections: All
 Search criteria All text fields: Plant Stress
We used “plant stress” as the search query term in “Patent Search” from Thomson Innovation (http://info.thomsoninnovation.com) for the period 1986–2015 and refined our search through the use of DWPI (Derwent World Patents Index) families. In order to distinguish between biotic and abiotic stresses, we chose query terms that we thought to be unique to each type of stress and used those terms in sub-searches subordinate to “plant stress”.
Retrieve DWPI family
Publication year filter: 1986-2015: 7659 DWPI families
Sub-search for biotic factors
Sub-search for abiotic factors
Sub-search results:
All text fields: (water* OR drought* OR flood* OR moisture* OR temperature* OR heat* OR cold* OR chill* OR freez* OR salt* OR salin* OR metal* OR pollut* )
Sub-search results:
All text fields: (pathogen* OR disease* OR virul*
OR infect*)
5496 DWPI families
7050 DWPI families

## Slide 3
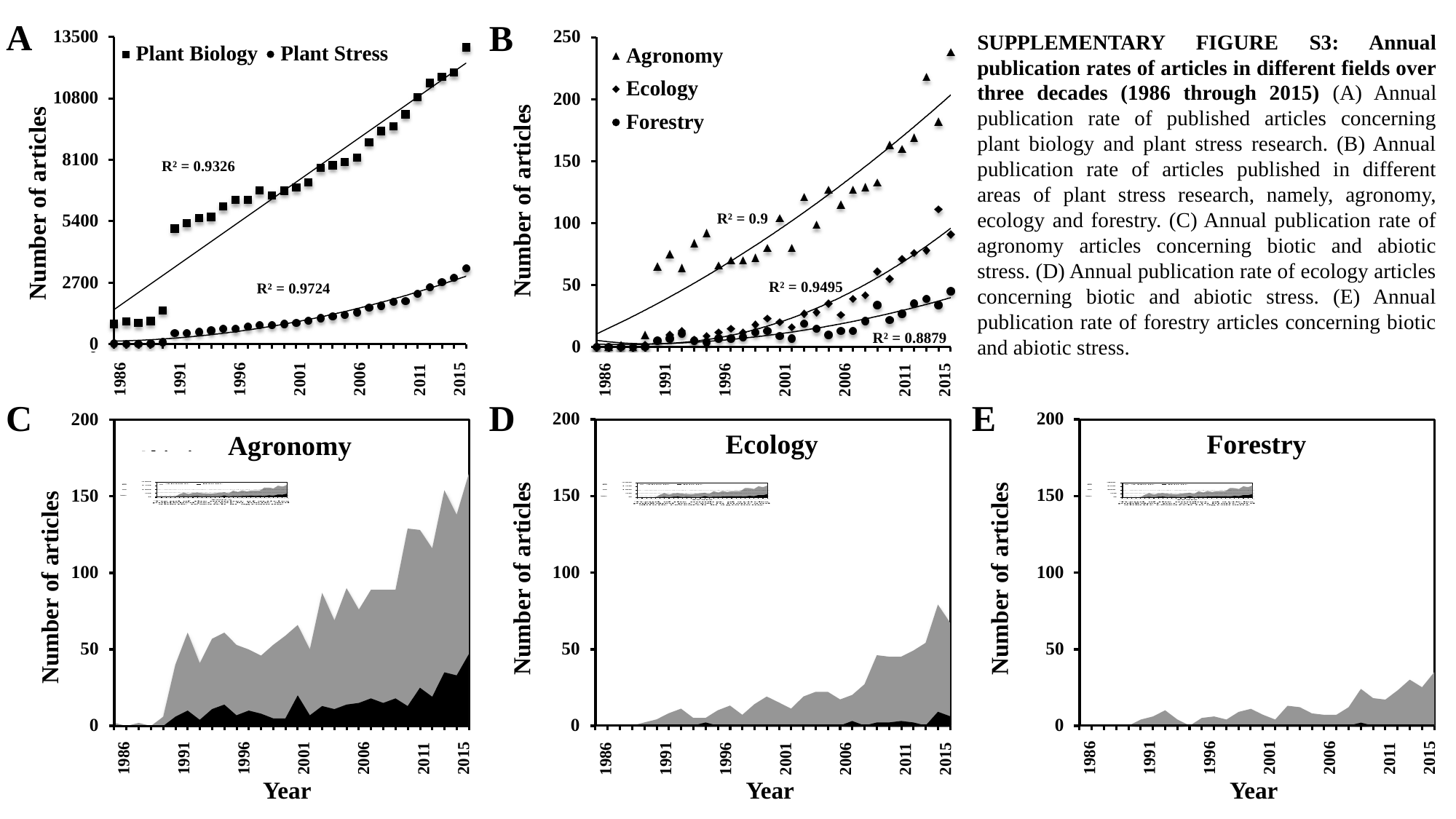

A
B
SUPPLEMENTARY FIGURE S3: Annual publication rates of articles in different fields over three decades (1986 through 2015) (A) Annual publication rate of published articles concerning plant biology and plant stress research. (B) Annual publication rate of articles published in different areas of plant stress research, namely, agronomy, ecology and forestry. (C) Annual publication rate of agronomy articles concerning biotic and abiotic stress. (D) Annual publication rate of ecology articles concerning biotic and abiotic stress. (E) Annual publication rate of forestry articles concerning biotic and abiotic stress.
Number of articles
Number of articles
1986
1991
1996
2001
2006
2011
2015
1986
1991
1996
2001
2006
2011
2015
C
D
E
Ecology
Forestry
Agronomy
Number of articles
Number of articles
Number of articles
1986
1991
1996
2001
2006
2011
2015
1986
1991
1996
2001
2006
2011
2015
1986
1991
1996
2001
2006
2011
2015
Year
Year
Year

## Slide 4
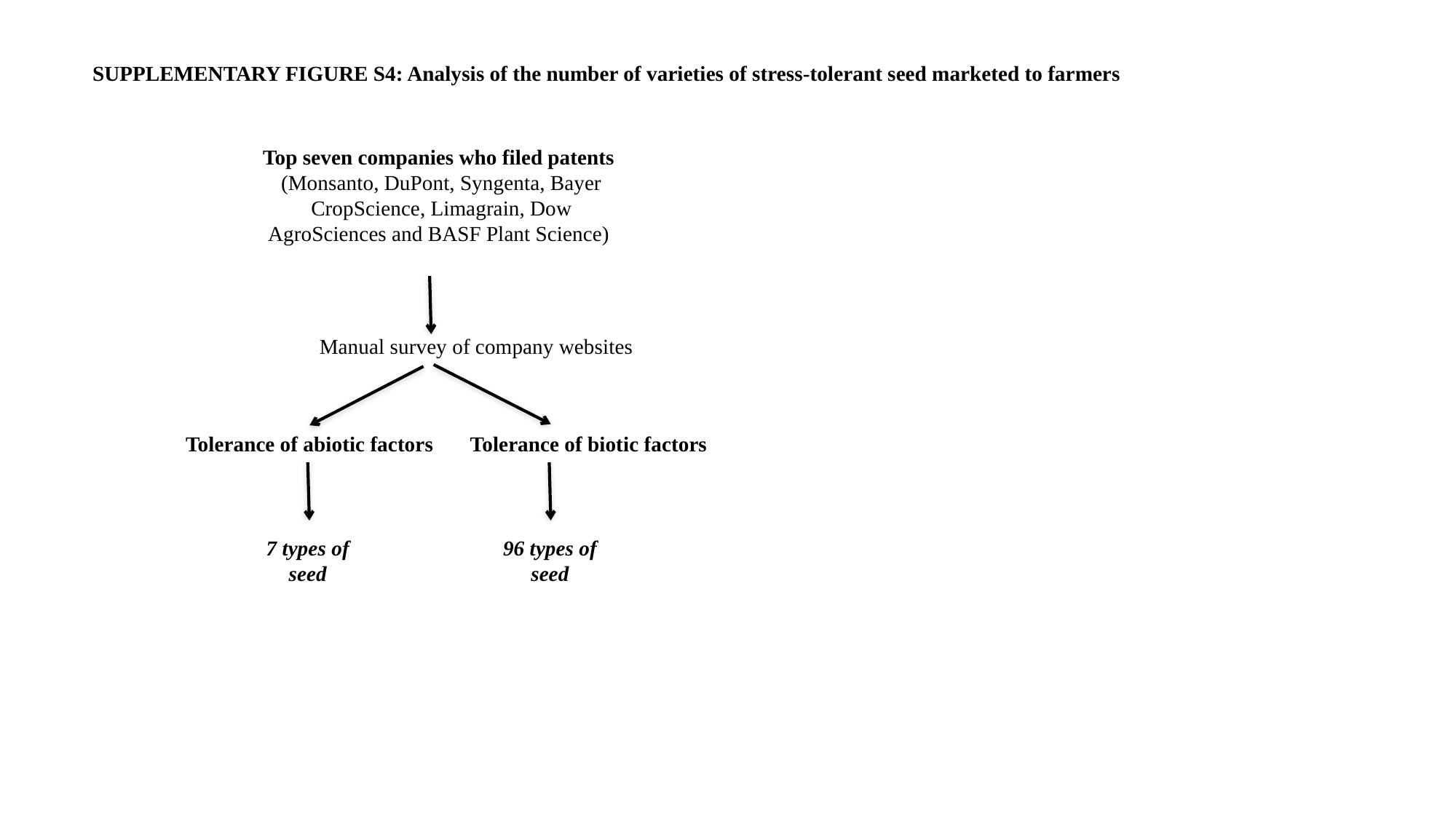

SUPPLEMENTARY FIGURE S4: Analysis of the number of varieties of stress-tolerant seed marketed to farmers
Top seven companies who filed patents
(Monsanto, DuPont, Syngenta, Bayer CropScience, Limagrain, Dow AgroSciences and BASF Plant Science)
Manual survey of company websites
Tolerance of biotic factors
Tolerance of abiotic factors
7 types of seed
96 types of seed
